# The lifelong maintenance of mesencephalic dopaminergic neurons by Nurr1 and engrailed

**DOI:** 10.1186/1423-0127-21-27

**Published:** 2014-04-01

**Authors:** Kambiz N Alavian, Sharmin Jeddi, Sahar I Naghipour, Pegah Nabili, Pawel Licznerski, Travis S Tierney

**Affiliations:** 1 Division of Brain Sciences, Department of Medicine, Imperial College, E508, Burlington Danes, Hammersmith Hospital, DuCane Road, London W12 0NN, UK; 2 The Bahá’í Institute for Higher Education (BIHE), Tehran, Iran; 3 Department of Internal Medicine, Endocrinology, Yale University, New Haven, CT, USA; 4 Department of Neurosurgery, Brigham and Women’s Hospital, Harvard Medical School, Boston, MA, USA

## Abstract

Specific vulnerability and degeneration of the dopaminergic neurons in the substantia nigra pars compacta of the midbrain is the pathological hallmark of Parkinson’s disease. A number of transcription factors regulate the birth and development of this set of neurons and some remain constitutively expressed throughout life. These maintenance transcription factors are closely associated with essential neurophysiological functions and are required ultimately for the long-term survival of the midbrain dopaminergic neurons. The current review describes the role of two such factors, Nurr1 and engrailed, in differentiation, maturation, and in normal physiological functions including acquisition of neurotransmitter identity. The review will also elucidate the relationship of these factors with life, vulnerability, degeneration and death of mesencephalic dopaminergic neurons in the context of Parkinson’s disease.

## Review

### Introduction

Parkinson’s disease (PD) is the second most prevalent neurodegenerative disorder, affecting 1-2% of the population over 65 and 3-5% of the people over 85 [1]. The disease initially manifests with the cardinal motor symptoms of rest tremor, bradykinesia, rigidity, and postural instability. Over time non-motor symptoms such as depression, constipation, pain, genitourinary problems, and sleep disorders [1,2] become prominent. The main pathological feature of PD is the degeneration of dopaminergic neurons in the substantia nigra pars compacta (SNpc). The other nearby mesencephalic dopaminergic (mesDA) neuronal populations within the ventral tegmental area (VTA) and retrorubral field (RRF) are less susceptible to degeneration and remain relatively unaffected during the course of the disease [3,4].

A wide variety of animal models have been developed to study the pathological outcomes of cell death in the ventral midbrain and to explore potential therapeutic targets that can stabilize or remedy the condition [5,6]. In recent years, characterization of differential gene expression profiles between the two main mesDA neuronal populations, VTA and SNpc, has been used to probe the question of relative susceptibility of neurons to environmental and genetic vulnerability [7]. Another approach to understand this susceptibility has been the study of the developmental cues that contribute directly or indirectly to differentiation of these phenotypes [8].

Based on these studies, we postulate that these neurons perpetually remain in a developmental state that is vulnerable to neurodegeneration because the critical period for naturally-occurring neuron death that occurs for most populations of neurons never completely closes for these unique cells in the ventral midbrain. This inherent vulnerability of mesDA neurons is a consequence of the unique expression profile of these cells which is controlled by a cascade of developmental transcription factors that are not only required for defining the regional identity of the midbrain and specification of mesDA neurons but also for the long-term survival and maintenance and normal physiological function. Several gain and loss of function studies in transgenic and mutant animals have demonstrated a strong connection between developmental factors and essential neuronal functions such as axon guidance, regulation of survival and cell death, defining the neurotransmitter phenotype, neuronal excitability and plasticity [8]. These studies have also established that the insufficiency in certain key transcription factors can lead to a neurodegenerative phenotype and subsequently to PD-like symptoms [9-12].

In this review we will discuss two transcription factors, engrailed and Nurr1, that are necessary for regulation of key events in the development of mesencephalic dopaminergic neurons as well as their survival and maintenance throughout life. In our discussion, we will highlight the role of these factors as key transcriptional regulators of differentiation, maturation and survival of mesDA neurons, the contribution of each factor to the normal physiological functions in postnatal mesDA neurons, as well as their possible connection with the vulnerability of neurons and etiology of Parkinson’s disease.

### Nurr1

Orphan nuclear receptor Nurr1 (nuclear receptor subfamily 4, group A, member 2; **NR4A2**) is a member of the steroid/thyroid hormone nuclear receptor and is related to a family of factors that modulate transcription in response to small lipophilic molecules. The transcriptional activity of these factors is regulated through the interaction of the ligand with the carboxy terminal ligand-binding domain of specific nuclear receptors [13]. Nurr1 transcription factor is encoded by an immediate early gene and is predominantly expressed in the brain [14,15]. Structurally, Nurr1 consists of a DNA binding domain, a ligand binding domain and two transcription activation function domains at the N- and C-termini (AF1 and AF2, respectively) [16,17]. The DNA binding domain of Nurr1 is highly conserved among the nuclear receptor family members and is comprised of two zinc finger modules. This domain is known to activate transcription through binding to an NGFI-B response element (NBRE) [18]. Nurr1 lacks a classical binding site for coactivators and the tight packing of side chains from hydrophobic residues in the ligand-binding domain prevents the molecule from having a ligand-binding cavity. The constitutive transcriptional activity of Nurr1, therefore, can be attributed to the canonical protein fold resembling the agonist-bound, transcriptionally active, ligand binding domains in nuclear receptors [19]. Nurr1 also activates transcription through heterodimerization with the retionid X receptor (RXR) and in response to the RXR ligands [20,21]. The AF1 and AF2 domains are also involved in co-factor recruitment and contribute to the constitutive transcriptional activation of Nurr1 [16,22].

In mice, the expression of Nurr1 can first be detected on embryonic day 10 (E10) in the ventral midbrain. Although the expression of the gene is reduced in the postnatal animals, it continues throughout life and has been shown to regulate several aspects of postmitotic development [10,23,24]. The role of Nurr1 in the survival and differentiation of mesDA neurons was initially discovered through mouse knockout studies, demonstrating loss of immunoreactivity for the rate-limiting enzyme in production of dopamine, tyrosine hydroxylase, in the mesDA neurons from Nurr1 knockout animals [25]. Later on further studies established that the neuroepithelial cells, that give rise to mesDA neurons, ventralize normally and differentiate into neurons. For example, they express mesDA neuron specific markers such as Pitx3, Ahd2 and engrailed1/2 and establish nigrostriatal axonal projections, but lack their dopaminergic phenotype [26-28]. Further examination of the transcriptional role of Nurr1 demonstrated that it is an upstream regulator of genes involved in the synthesis, packaging, transport and reuptake of dopamine [29,30]. For example, Nurr1 has been shown to induce the expression of tyrosine hydroxylase (TH) by directly transactivating the promoter of TH [31]. Regulation of the dopamine transporter (DAT) gene is another key function of Nurr1 in determining the neurotransmitter phenotype of mesDA neurons. This effect of Nurr1 seems to be independent of its classic heterodimerization partner, retinoid X receptor. The expression of TH and DAT is regulated by the high affinity binding of Nurr1 to an extended half-hormone response element, NGF1-B responsive element (NBRE), in their 5′-untranslated regions [32,33]. Finally, other studies have shown that Nurr1 is involved in conversion of L-DOPA to DA and packaging of DA into synaptic vesicles by regulating the expression of aromatic L-amino acid decarboxylase (AADC) and vesicular monoamine transporter-2 (VMAT2), respectively [30].

In addition to transcriptional regulation of the genes involved in dopamine production and trafficking, Nurr1 is also a key convert factor in several survival and maintenance pathways. Nurr1 regulates the rearranged in transfection (Ret) gene from the earliest stages in embryonic development. Ret tyrosine kinase is the high-affinity ligand-binding component of the glial cell line derived neurotrophic factor (GDNF) receptor complex that is attached to the cell surface via a glycosyl phosphatidylinositol anchor [34]. GDNF has been shown to protect mesDA neurons against the developmental waves of apoptosis, neurotoxic insults and cell death [35-38]. Upon GDNF binding, autophosphorylation of the tyrosine domains of Ret triggers activation of several pathways including PI3K, MAPK which are required for neuronal survival and neurite outgrowth [39]. Interestingly, one of the downstream phosphorylation targets of the GDNF signaling pathway is the cAMP response element binding (CREB) protein, which is known to directly regulate the expression of Nurr1 by binding to its promoter [40].

**Figure 1:**
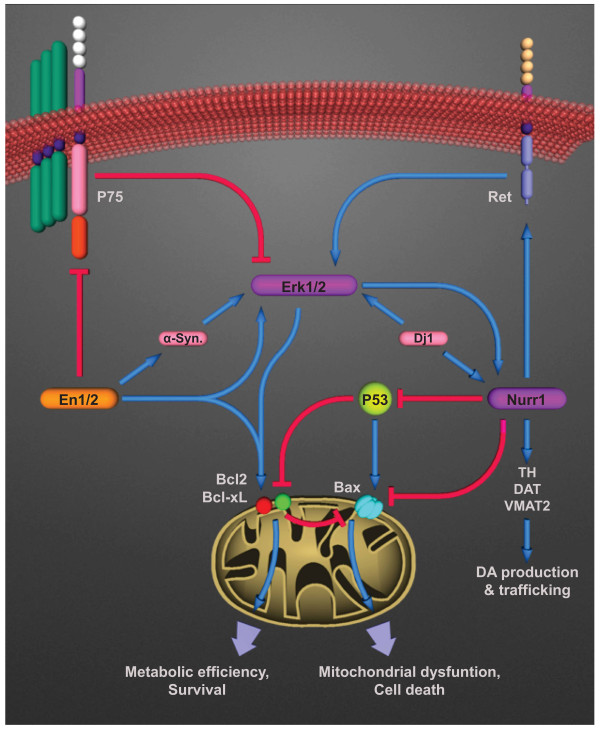
**Signaling pathways involved in regulation of cellular survival and death by engrailed and Nurr1 in mesencephalic dopaminergic neurons.** Transcriptional regulation of several survival/death-regulating genes including the pan-neurotrophin receptor P75 and protooncogene Ret, as well as modulation of downstream survival mechanisms, such as MAPK (Erk1/2) by engrailed and Nurr1 leads to the long-term regulation of survival and death of mesDA neurons. Down-regulation of P75 expression by the engrailed genes results in disinhibition of Erk1/2, resulting in survival of mesDA neurons. The effect is similar to the Ret-mediated activation of Erk1/2 by Nurr1. The survival and degeneration pathways regulated by engrailed and Nurr1 converge on mitochondria and are mediated by the anti- and pro-apoptotic members of the Bcl-2 family, Bcl2, Bcl-xL and Bax.

Homozygous Nurr1 knockout pups die shortly after birth following degeneration of mesDA neurons [25-27]. However, Nurr1 haploinsufficient animals show no significant loss of mesDA neuron in number or motor function until late in life. The aging (>15 months old) Nurr1+/− mice have a decrease in the number of their nigral mesDA neurons and their locomotor activities, correlated with the reduced mesolimbic and mesocortical dopamine levels [9,23]. These animals also display increased vulnerability and cell death in response to neurotoxic insult [9,41]. The increased neuronal degeneration and death in these animals is likely to be due to mitochondrial dysfunction and the opening of the mitochondrial permeability transition pore [42]. Transcriptional regulation of the pro-and anti-apoptotic members of the Bcl-2 mitochondrial family of proteins and interaction with the P53 tumor suppressor protein by Nurr1 are responsible for regulation of mitochondrial survival and death (Figure 1) [43]. The conditional knockout studies have further elucidated the role of Nurr1 in the survival and maintenance of mesDA neurons in the adult animals, showing that Nurr1-deficiency in maturing neurons results in a rapid loss of striatal dopamine, loss of mesDA specific markers and degeneration [10]. Taken together, these studies show that Nurr1 regulates both the phenotypic expression and survival of mesDA neurons in mice.

Given the neuoprotective role of Nurr1 demonstrated in animal models and its decreased or diminished expression levels in PD patients [44,45], a number of human genetic studies of the Nurr1 gene were undertaking to identify potential variants that may increase the risk of developing PD. Although the results from the studies within the past decade have failed to establish the mutations in Nurr1 as a cause or direct risk factor for PD, higher incidence of several mutations within the coding and non-coding regions of Nurr1 have been observed in familial and sporadic cases of the disease [46-49].

A number of these mutations have been associated with a marked decrease in the level of Nurr1 expression and subsequent reduction in the expression of TH [46]. The evidence from these studies accompanied by the results of the postmortem expression analysis in the PD patient brains suggest dysregulation of Nurr1 as a contributing factor to the onset and progression of neurodegeneration during the course of PD.

### The engrailed transcription factors

The engrailed genes belong to the family of homeobox transcription factors, containing a highly conserved DNA binding, helix-turn-helix, homeodomain protein fold [50]. The morphological significance of the engrailed was first described in drosophila, where the autosomal mutant displays a long cleft of the thorax and irregular venation of the wings [51]. In addition to its early role in body segmentation, engrailed homologs in various annelid, mollusks, chordate and arthropod species has revealed a separate major function in neurogenesis [52-54]. Interestingly, homologs of engrailed are conserved among different phyla, to the degree that replacement of the mouse engrailed-1 gene by its paralog En2 or by the drosophila engrailed homolog will preserve its function in development and survival [55,56].

The engrailed genes have two distinct functions during early and late development. The two paralogs of engrailed, En1 and En2, start their expression around E8 in mice in a broad region at the midbrain/hindbrain border, known as the isthmic organizer [57]. During this period, engrailed is involved in the induction of mesDA neurons by maintaining expression of Fgf8 in this region [58-60]. En1/2 directly regulate the expression of Fgf8 through interaction with a DNA-binding intronic fragment on Fgf8 [61]. In En1/2 deficient embryos the domain of expression of Fgf8 in the isthmus is reduced and leads an incomplete induction of mesDA neurons in the midbrain. The specific expression of the engrailed genes in mesDA neurons starts after the neurons are induced and become postmitotic, between E11.5 and E14 in mice [62]. After this point, expression of both engrailed genes is required for continued survival of these neurons.

Mouse knockout studies have shown that there is a direct correlation between the dose of expression of engrailed and its survival effect in mesDA neurons. Mice lacking all four alleles of the two transcription factors (En1−/−;En2−/−) show the most severe phenotype. In these mice, the induction of dopaminergic neurons in the ventral midbrain is affected, the differentiated postmitotic mesDA neurons display significant developmental defects including a lack of axonal outgrowth and eventually die between E12 and E14, matching the timeframe of the expression of engrailed genes in wildtype mesDA neurons [62,63]. The postmitotic survival effect of En1 is more prominent than that of En2, since the mice lacking En2 show less severe phenotypes than the En1−/− animals. All En1−/− animals (regardless of the expression of En2) exhibit an abnormal distribution of dopaminergic neurons in ventral midbrain and die at birth. En1+/+;En2−/− mice, however, are viable and fertile and show no obvious deficiencies with regard to their dopaminergic neurons [62,64]. The most interesting phenotype among the engrailed-deficient animals is that of the mice heterozygous for En1 and homozygous null for En2 (En1+/−;En2−/−) where the development of mesDA neurons seems to be normal until just after birth when 70% of the neurons are lost within the first three 3 postnatal months. This specific nigral dopaminergic cell loss is accompanied by diminished dopamine levels and release within the dorsal striatum, leading to a marked decrease in locomotion, an increase in freezing episodes and weight loss.

Engrailed regulates development, survival and maintenance of mesDA neurons through the cooperative action of multiple molecular pathways. Cell death in absence of engrailed is mediated by at least two molecular pathways, involving the pan-neurotrophin receptor, P75NTR and the mitochondria. The increase in expression of the neurotrophin receptor P75 is a common phenomenon observed in neuronal injury and naturally-occurring neuron death [65]. Similarly, engrailed deficiency causes an increase in the expression of P75 resulting in downregulation of the mitogen activated protein kinase (MAPK) survival pathways and deactivation of the extracellular regulated kinases Erk1/2 [66]. A second survival mechanism regulated by the expression of the engrailed genes affects the mitochondria. Similar to the Nurr1 haploinsufficient cells, the mesDA neurons heterozygous for En1 (either in presence or absence of En2) are more prone to mitochondrial insult and cell death induced by inhibition of the complex-I of the electron transport chain than their wildtype counterparts [66]. The dose-dependent survival role of the engrailed genes against mitochondrial instability is suggestive of another link between engrailed and the etiology of PD, since mitochondrial dysfunction has been implicated as one of the most prominent molecular mechanisms in the pathoetiology of Parkinson’s disease [66-69].

These studies on the engrailed knockout animals spurred a search for mutant genes in humans with Parkinson’s disease. These studies have established a significant association between sporadic PD and single nucleotide polymorphisms (SNPs) in an intronic region of En1, on the 3′ downstream region of En1 and the promoter region of En2 [70-72].

## Conclusions

Degeneration of dopaminergic neurons in the substantia nigra pars compacta is pathological hallmark of PD that is responsible for most of the motor symptoms of the disease. The inherent vulnerability of this neuronal population, as a root cause for degeneration, is attributed to the distinct gene expression profile of mesDA neurons in this region, which sets them apart from the neighboring neurons in the ventral tegmental area [73-75]. There is ample evidence suggesting that this basic physiological feature (i.e. vulnerability to degeneration) of the neurons and the underlying specific gene expression profile are defined very early on during the time that the cells assume their positional identity, become postmitotic and express their dopaminergic phenotype, even before the onset of axonal outgrowth. Some of the most compelling and direct evidence in support of this argument comes from the studies on the mice lacking the homeodomain transcription factor Pitx3, where the development of the neurons is halted, resulting in cell death only in the substantia nigra around E12 [76]. The engrailed transcription factors and Nurr1 begin their expression in mesDA neurons during the same time and, to a large extent, define the molecular identity of these neurons throughout life.

Given the gene-dose-dependent loss of mesDA neurons in the engrailed deficient mice and the severe mesDA-specific defects in the Nurr1 knockout animals, it can be concluded that the main role of these two factors beyond this critical period (E12-E14 in mice) is to protect the nigral dopaminergic neurons by maintaining the survival cues that are most active during the developmental and early stages of the life of mesDA neurons. The insights into the transcriptional regulation of survival by these factors, therefore, may provide an insight into the core issue of the specific vulnerability of nigral dopaminergic neurons. In recent years, a number of studies have revealed some of the downstream transcriptional targets and survival mechanisms that converge at the level of mitochondria and downstream of growth factors [66,67]. The expression of engrailed genes indirectly determines the level of phosphorylation of two mitogen activated protein kinase (MAPK) family members, Erk1/2, which act as mediators of survival, proliferation and differentiation in response to growth factors [66]. Erk1/2 as well as the other member of the MAPK family, Erk5, are known to contribute to the expression of TH, differentiation as well as survival of mesDA neurons by elevating the transcriptional activity of Nurr1 (Figure 1) [77,78]. Erk1/2 can also cause phosphorylation, nuclear localization and activation of Nurr1, which in turn affects the expression levels of tyrosine hydroxylase. This mechanism is related to the expression of the autosomal recessive early onset PD gene DJ-1 (Figure 1) [79].

En1/2 and Nurr1 expression also prevents mitochondrial impairment, a well described pathogenic phenomenon in Parkinson’s disease, by inhibiting neurotoxic insult and the mitochondrial pathway of apoptosis, through regulation of the pro- and anti-apoptotic member of Bcl-2 family (Figure 1) [43,66,80]. This function of the two transcription factors is likely to prevent the cells from undergoing mitochondrial permeability transition, which occurs in response to death stimuli. While the opening of the mitochondrial permeability transition pore is generally considered an acute pathological phenomenon, under certain conditions such as metabolic stress, it is plausible that subtle changes in the mitochondrial membrane potential or uncoupling of the mitochondrial inner membrane would result in chronic neurodegeneration. This mechanism is more likely to be the target for the long-term protective effects of engrailed and Nurr1. The molecular nature of this protective function remains to be determined.

## Competing interests

The authors declare that they have no competing interests.

## Authors’ contributions

KNA wrote the manuscript; SJ, SIN, PN, PL and TST critically read and revised the manuscript. All authors have read and approved the final manuscript.
